# Supplementary material for: The successful and safe conversion of joint arthroplasty to same-day surgery: A necessity after the COVID-19 pandemic
Source: PLoS One. 2023 Nov 27;18(11):e0290135. doi: 10.1371/journal.pone.0290135 (PMC10681212; doi:10.1371/journal.pone.0290135)
Supplement: S1 File — (DOCX) [file pone.0290135.s001.docx]

# Fig S1: Number of elective arthroplasties by setting over time


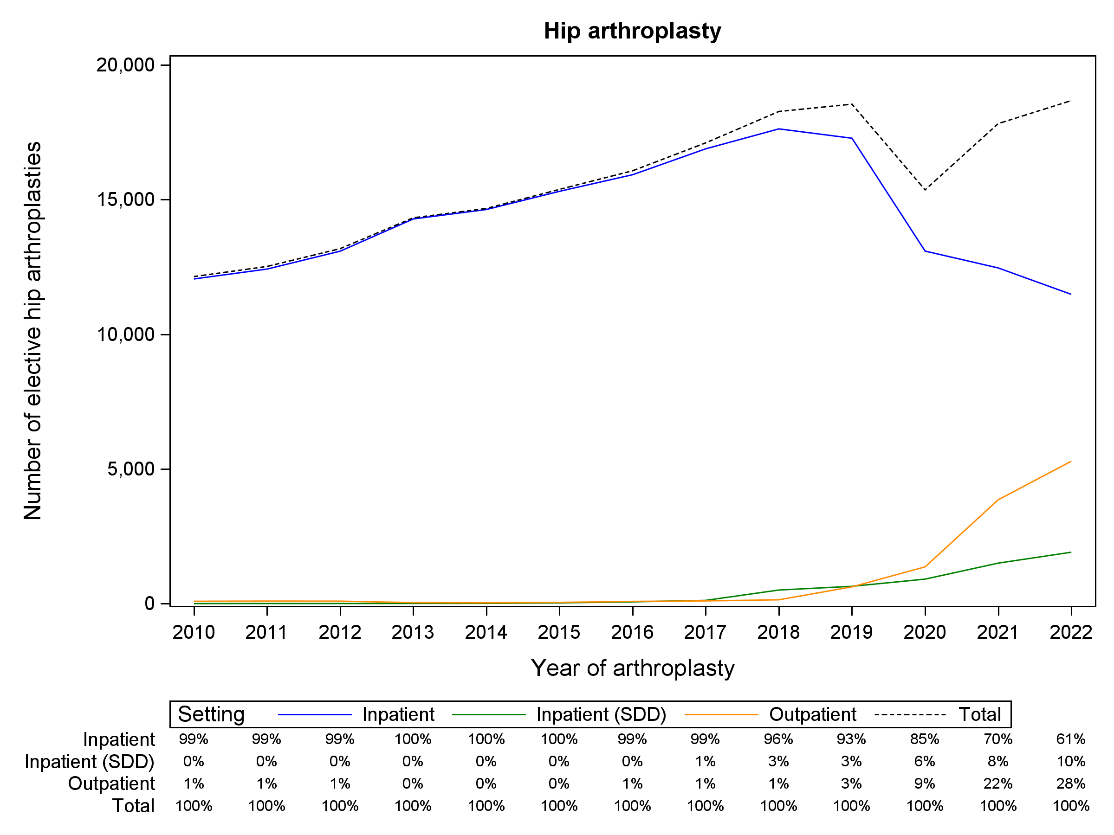

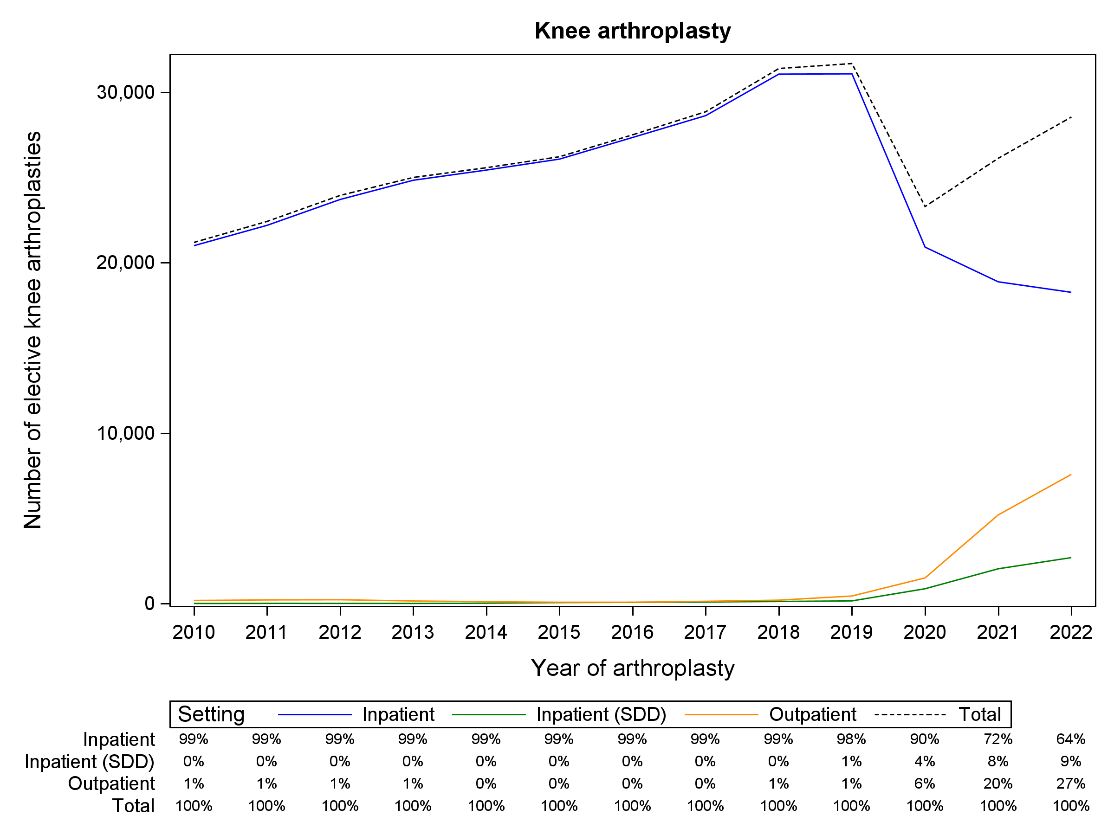


**S1 Fig. Number of elective arthroplasties by setting over time.** Number of elective hip and knee arthroplasties over time by setting as defined using outpatient records (National Ambulatory Care Reporting System) and inpatient records (Discharge Abstract Database). The inpatient records were split into those where the discharge date was the same as the surgery date (same-day discharge; SDD) or after the surgery date.

# Fig S2: Observed versus expected number of hip and knee arthroplasties per week


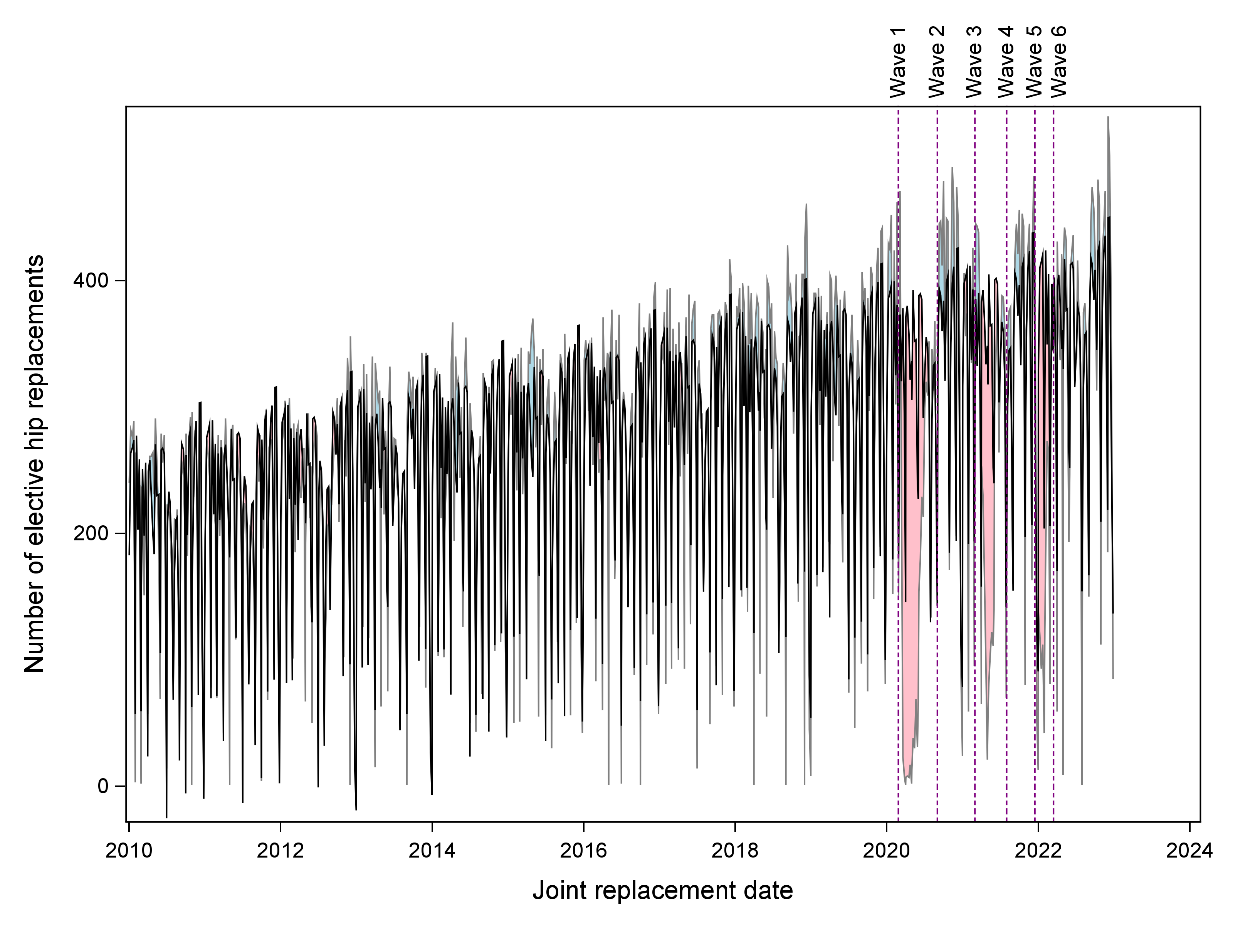

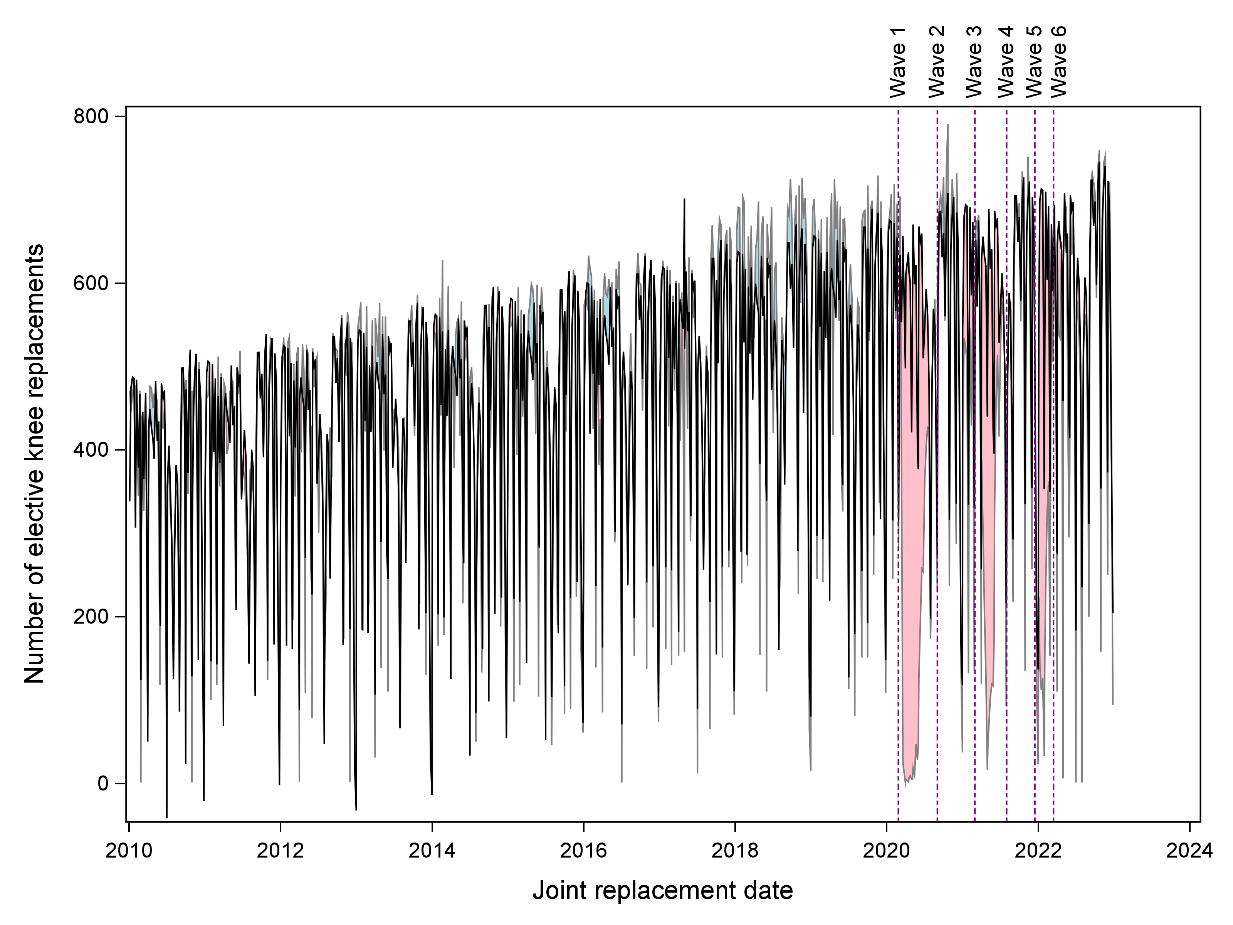


**S2 Fig. Observed versus expected number of hip and knee arthroplasties per week.** Expected number shown as black line; observed number is shown as deviations from that line. If more than expected, the shading between observed and expected is blue; if less than expected the shading between observed and expected is red.

# Table S1: Administrative codes

| CCI code | Description | N |
| --- | --- | --- |
| Hip arthroplasty – partial | | |
| 1SQ53LAPM | Implantation of internal device, pelvis uncemented prosthetic device, single component [e.g. cup] | 213 |
| 1SQ53LAPMA | Implantation of internal device, pelvis using bone autograft (uncemented) prosthetic device, single component [e.g. cup] | 69 |
| 1SQ53LAPMK | Implantation of internal device, pelvis using bone homograft (uncemented) prosthetic device, single component [e.g. cup] | 21 |
| 1SQ53LAPMN | Implantation of internal device, pelvis using synthetic tissue [e.g. bone cement or paste] prosthetic device, single component [e.g. cup] | 83 |
| 1SQ53LAPMQ | Implantation of internal device, pelvis using combined sources of tissue [e.g. bone graft, cement/paste] prosthetic device, single component [e.g. cup] | 34 |
| 1SQ53LAPR | Implantation of internal device, pelvis uncemented partial component [e.g. acetabular liner (insert) alone] | <6 |
| 1VA53LAPM | Implantation of internal device, hip joint open approach (direct lateral, posterolateral, posterior, transgluteal) uncemented single component prosthetic device [femoral] | 26164 |
| 1VA53LAPMA | Implantation of internal device, hip joint open approach (direct lateral, posterolateral, posterior, transgluteal) using bone autograft [uncemented] single component prosthetic device [femoral] | 4604 |
| 1VA53LAPMK | Implantation of internal device, hip joint open approach (direct lateral, posterolateral, posterior, transgluteal) using bone homograft [uncemented] single component prosthetic device [femoral] | 202 |
| 1VA53LAPMN | Implantation of internal device, hip joint open approach (direct lateral, posterolateral, posterior, transgluteal) using synthetic material (e.g. bone paste, cement) single component prosthetic device [femoral] | 17746 |
| 1VA53LAPMQ | Implantation of internal device, hip joint open approach (direct lateral, posterolateral, posterior, transgluteal) using combined sources of tissue (e.g. bone graft, cement, paste) single component prosthetic device [femoral] | 1639 |
| 1VA53LASLN | Implantation of internal device, hip joint open approach (direct lateral, posterolateral, posterior, transgluteal) using synthetic material (e.g. bone paste, cement) cement spacer [temporary, impregnated with antibiotics] | 1531 |
| 1VA53LLPM | Implantation of internal device, hip joint open anterior (muscle sparing) approach (anterolateral, direct anterior) uncemented single component prosthetic device [femoral] | 878 |
| 1VA53LLPMA | Implantation of internal device, hip joint open anterior (muscle sparing) approach (anterolateral, direct anterior) using bone autograft [uncemented] single component prosthetic device [femoral] | 183 |
| 1VA53LLPMK | Implantation of internal device, hip joint open anterior (muscle sparing) approach (anterolateral, direct anterior) using bone homograft [uncemented] single component prosthetic device [femoral] | <6 |
| 1VA53LLPMN | Implantation of internal device, hip joint open anterior (muscle sparing) approach (anterolateral, direct anterior) using synthetic material (e.g. bone paste, cement) single component prosthetic device [femoral] | 612 |
| 1VA53LLPMQ | Implantation of internal device, hip joint open anterior (muscle sparing) approach (anterolateral, direct anterior) using combined sources of tissue (e.g. bone graft, cement, paste) single component prosthetic device [femoral] | 72 |
| 1VA53LLSLN | Implantation of internal device, hip joint open anterior (muscle sparing) approach (anterolateral, direct anterior) using synthetic material (e.g. bone paste, cement) cement spacer [temporary, impregnated with antibiotics] | 48 |
| 1VA53PNPM | Implantation of internal device (robotic open approach), pelvis using bone autograft (uncemented) prosthetic device, single component [e.g. cup] | <6 |
| 1VA53PNPMA | Implantation of internal device (robotic open approach), pelvis uncemented prosthetic device, single component [e.g. cup] | <6 |
| 1VA53PNPMN | Single component prosthetic hip robotic open approach with synthetic material (e.g. bone paste, cement) | <6 |
|  |  |  |
| Hip arthroplasty – total | | |
| 1SQ53LAPN | Implantation of internal device, pelvis uncemented prosthetic device, dual component [e.g. cup with liner] | 136 |
| 1SQ53LAPNA | Implantation of internal device, pelvis using bone autograft (uncemented) prosthetic device, dual component [e.g. cup with liner] | 87 |
| 1SQ53LAPNK | Implantation of internal device, pelvis using bone homograft (uncemented) prosthetic device, dual component [e.g. cup with liner] | 28 |
| 1SQ53LAPNN | Implantation of internal device, pelvis using synthetic tissue [e.g. bone cement or paste] prosthetic device, dual component [e.g. cup with liner] | 69 |
| 1SQ53LAPNQ | Implantation of internal device, pelvis using combined sources of tissue [e.g. bone graft, cement/paste] prosthetic device, dual component [e.g. cup with liner] | 67 |
| 1VA53LAPN | Implantation of internal device, hip joint open approach (direct lateral, posterolateral, posterior, transgluteal) uncemented dual component prosthetic device [femoral with acetabular] | 66368 |
| 1VA53LAPNA | Implantation of internal device, hip joint open approach (direct lateral, posterolateral, posterior, transgluteal) using bone autograft [uncemented] dual component prosthetic device [femoral with acetabular] | 94877 |
| 1VA53LAPNK | Implantation of internal device, hip joint open approach (direct lateral, posterolateral, posterior, transgluteal) using bone homograft [uncemented] dual component prosthetic device [femoral with acetabular] | 2514 |
| 1VA53LAPNN | Implantation of internal device, hip joint open approach (direct lateral, posterolateral, posterior, transgluteal) using synthetic material (e.g. bone paste, cement) dual component prosthetic device [femoral with acetabular] | 12686 |
| 1VA53LAPNQ | Implantation of internal device, hip joint open approach (direct lateral, posterolateral, posterior, transgluteal) using combined sources of tissue (e.g. bone graft, cement, paste) dual component prosthetic device [femoral with acetabular] | 10089 |
| 1VA53LLPN | Implantation of internal device, hip joint open anterior (muscle sparing) approach (anterolateral, direct anterior) uncemented dual component prosthetic device [femoral with acetabular] | 8758 |
| 1VA53LLPNA | Implantation of internal device, hip joint open anterior (muscle sparing) approach (anterolateral, direct anterior) using bone autograft [uncemented] dual component prosthetic device [femoral with acetabular] | 12291 |
| 1VA53LLPNK | Implantation of internal device, hip joint open anterior (muscle sparing) approach (anterolateral, direct anterior) using bone homograft [uncemented] dual component prosthetic device [femoral with acetabular] | 173 |
| 1VA53LLPNN | Implantation of internal device, hip joint open anterior (muscle sparing) approach (anterolateral, direct anterior) using synthetic material (e.g. bone paste, cement) dual component prosthetic device [femoral with acetabular] | 868 |
| 1VA53LLPNQ | Implantation of internal device, hip joint open anterior (muscle sparing) approach (anterolateral, direct anterior) using combined sources of tissue (e.g. bone graft, cement, paste) dual component prosthetic device [femoral with acetabular] | 899 |
| 1VA53PNPN | Dual component prosthetic hip robotic open approach | <6 |
| 1VA53PNPNA | Dual component prosthetic hip robotic open approach using bone autograft (uncemented) prosthetic device, dual component [e.g. cup with liner] | <6 |
| 1VA53PNPNN | Dual component prosthetic hip robotic open approach with synthetic material | <6 |
|  |  |  |
| Knee arthroplasty – partial | | |
| 1VG53LAPM | Implantation of internal device, knee joint uncemented single component prosthetic device | 3499 |
| 1VG53LAPMA | Implantation of internal device, knee joint with bone autograft single component prosthetic device | 185 |
| 1VG53LAPMK | Implantation of internal device, knee joint with bone homograft single component prosthetic device | 12 |
| 1VG53LAPMN | Implantation of internal device, knee joint with synthetic material (e.g. bone paste, cement) single component prosthetic device | 2169 |
| 1VG53LAPMQ | Implantation of internal device, knee joint with combined sources of tissue (e.g. bone graft, cement, paste) single component prosthetic device | 536 |
| 1VG53LAPR | Implantation of internal device, knee joint uncemented partial component [e.g. tibial liner (insert) alone] | 1441 |
| 1VG53LASLN | Implantation of internal device, knee joint with synthetic material (e.g. bone paste, cement) cement spacer [temporary] [impregnated with antibiotics] | 2386 |
| 1VP53LAPM | Implantation of internal device, patella of uncemented single-component [patella only] prosthetic device using open approach | 109 |
| 1VP53LAPMN | Implantation of internal device, patella of cemented single-component [patella only] prosthetic device using open approach | 783 |
|  |  |  |
| Knee arthroplasty – total | | |
| 1VG53LAPN | Implantation of internal device, knee joint uncemented using dual component prosthetic device | 5725 |
| 1VG53LAPNA | Implantation of internal device, knee joint with bone autograft dual component prosthetic device | 9264 |
| 1VG53LAPNK | Implantation of internal device, knee joint with bone homograft dual component prosthetic device | 135 |
| 1VG53LAPNN | Implantation of internal device, knee joint with synthetic material (e.g. bone paste, cement) dual component prosthetic device | 33246 |
| 1VG53LAPNQ | Implantation of internal device, knee joint with combined sources of tissue (e.g. bone graft, cement, paste) dual component prosthetic device | 62009 |
| 1VG53LAPP | Implantation of internal device, knee joint uncemented tri component prosthetic device | 7235 |
| 1VG53LAPPA | Implantation of internal device, knee joint with bone autograft tri component prosthetic device | 4025 |
| 1VG53LAPPK | Implantation of internal device, knee joint with bone homograft tri component prosthetic device | 175 |
| 1VG53LAPPN | Implantation of internal device, knee joint with synthetic material (e.g. bone paste, cement) tri component prosthetic device | 74197 |
| 1VG53LAPPQ | Implantation of internal device, knee joint with combined sources of tissue (e.g. bone graft, cement, paste) tri component prosthetic device | 125240 |
| 1VP53LAPN | Implantation of internal device, patella of uncemented dual-component [patellofemoral] prosthetic device using open approach | 70 |
| 1VP53LAPNN | Implantation of internal device, patella of cemented dual-component [patellofemoral] prosthetic device using open approach | 697 |
| ^a^ assigned as partial or total based on digits 8 and 9 (PN – dual component; PM – single component)  CCI – Canadian Classification of Interventions | | |

# Table S2: Comorbidity codes

| **Condition** | **International Classification of Diseases (10^th^ revision)** |
| --- | --- |
| Myocardial infarction | 'I21' 'I22' 'I252' |
| Congestive heart failure | 'I43' 'I50' 'I099' 'I110' 'I130' 'I132' 'I255' 'I420' 'I425' 'I426' 'I427' 'I428' 'I429' 'P290' |
| Peripheral vascular disease | 'I70' 'I710' 'I711' 'I712' 'I713' 'I714' 'I715' 'I716' 'I717' 'I718' 'I719' 'I731' 'I738' 'I739' 'I771' 'I790' 'I792' 'K551' 'K558' 'K559' 'Z958' 'Z959' |
| Cerebrovascular disease | 'G45' 'G46' 'I60' 'I61' 'I62' 'I63' 'I64' 'I65' 'I66' 'I67' 'I68' 'I69' 'H340' |
| Dementia | 'F00' 'F01' 'F02' 'F03' 'G30' 'F051' 'G311' |
| Chronic pulmonary disease | 'J40' 'J41' 'J42' 'J43' 'J44' 'J45' 'J46' 'J47' 'J60' 'J61' 'J62' 'J63' 'J64' 'J65' 'J66' 'J67' 'I278' 'I279' 'J684' 'J701' 'J703' |
| Connective tissue/rheumatic disease | 'M32' 'M33' 'M34' 'M315' 'M351' 'M353' 'M360' (M05' 'M06' were removed) |
| Peptic ulcer disease | 'K25' 'K26' 'K27' 'K28' |
| Mild liver disease | 'B18' 'K73' 'K74' 'K700' 'K701' 'K702' 'K703' 'K709' 'K717' 'K713' 'K714' 'K715' 'K760' 'K762' 'K763' 'K764' 'K768' 'K769' 'Z944' |
| Diabetes without complications | 'E100' 'E101' 'E106' 'E108' 'E109' 'E110' 'E111' 'E116' 'E118' 'E119' 'E120' 'E121' 'E126' 'E128' 'E129' 'E130' 'E131' 'E136' 'E138' 'E139' 'E140' 'E141' 'E146' 'E148' 'E149' |
| Diabetes with complications | 'E102' 'E103' 'E104' 'E105' 'E107' 'E112' 'E113' 'E114' 'E115' 'E117' 'E122' 'E123' 'E124' 'E125' 'E127' 'E132' 'E133' 'E134' 'E135' 'E137' 'E142' 'E143' 'E144' 'E145' 'E147' |
| Paraplegia or hemiplegia | 'G81' 'G82' 'G041' 'G114' 'G801' 'G802' 'G830' 'G831' 'G832' 'G833' 'G834' 'G839' |
| Renal disease | 'N18' 'N19' 'N052' 'N053' 'N054' 'N055' 'N056' 'N057' 'N250' 'I120' 'I131' 'N032' 'N033' 'N034' 'N035' 'N036' 'N037' 'Z490' 'Z491' 'Z492' 'Z940' 'Z992' |
| Primary cancer | 'C00' 'C01' 'C02' 'C03' 'C04' 'C05' 'C06' 'C07' 'C08' 'C09' 'C10' 'C11' 'C12' 'C13' 'C14' 'C15' 'C16' 'C17' 'C18' 'C19' 'C20' 'C21' 'C22' 'C23' 'C24' 'C25' 'C26' 'C30' 'C31' 'C32' 'C33' 'C34' 'C37' 'C38' 'C39' 'C40' 'C41' 'C43' 'C45' 'C46' 'C47' 'C48' 'C49' 'C50' 'C51' 'C52' 'C53' 'C54' 'C55' 'C56' 'C57' 'C58' 'C60' 'C61' 'C62' 'C63' 'C64' 'C65' 'C66' 'C67' 'C68' 'C69' 'C70' 'C71' 'C72' 'C73' 'C74' 'C75' 'C76' 'C81' 'C82' 'C83' 'C84' 'C85' 'C88' 'C90' 'C91' 'C92' 'C93' 'C94' 'C95' 'C96' 'C97' |
| Moderate or severe liver disease | 'K704' 'K711' 'K721' 'K729' 'K765' 'K766' 'K767' 'I850' 'I859' 'I864' 'I982' |
| Metastatic cancer | 'C77' 'C78' 'C79' 'C80' |
| Human immunodeficiency virus | 'B20' 'B21' 'B22' 'B24' |

# Table S3: Drug Identification Numbers for defining diabetes as a comorbidity from the Ontario Drug Benefits database (age 65+)

| **DIN** | **Generic Name** | **brand name, strength, dosage, form** |
| --- | --- | --- |
| 02257726 | Metformin HCL | ACT Metformin Act Metformin 500mg Tab |
| 02167786 | Metformin HCL | Apo-Metformin 500mg Tab |
| 02438275 | Metformin HCL | Auro-Metformin 500mg Tab |
| 02380196 | Metformin HCL | Jamp-Metformin 500mg Tab |
| 02353377 | Metformin HCL | Metformin 500mg Tab |
| 02385341 | Metformin HCL | Metformin FC 500mg Tab |
| 02388766 | Metformin HCL | Mint-Metformin 500mg Tab |
| 02045710 | Metformin HCL | Novo-Metformin 500mg Tab |
| 02223562 | Metformin HCL | PMS-Metformin 500mg Tab |
| 02520303 | Metformin HCL | PMSC-Metformin 500mg Tab |
| 02269031 | Metformin HCL | Ran-Metformin 500mg Tab |
| 02242974 | Metformin HCL | Ratio-Metformin 500mg Tab |
| 02246820 | Metformin HCL | Sandoz Metformin FC 500mg Tab |
| 02379767 | Metformin HCL | Septa-Metformin 500mg Tab |
| 02099233 | Metformin HCL | Glucophage 500mg Tab |
| 02333856 | Metformin & Sitagliptin | Janumet 500mg & 50mg Tab |
| 02333864 | Metformin & Sitagliptin | Janumet 850mg & 50mg Tab |
| 02333872 | Metformin & Sitagliptin | Janumet 1000mg & 50mg Tab |
| 02416786 | Metformin & Sitagliptin | Janumet XR 500mg & 50mg ER Tab |
| 02416794 | Metformin & Sitagliptin | Janumet XR 1000mg & 50mg ER Tab |
| 02416808 | Metformin & Sitagliptin | Janumet XR 1000mg & 100mg ER Tab |
| 02403250 | Linagliptin & Metformin | Jentadueto 2.5mg & 500mg Tab |
| 02403269 | Linagliptin & Metformin | Jentadueto 2.5mg & 850mg Tab |
| 02403277 | Linagliptin & Metformin | Jentadueto 2.5mg & 1000mg Tab |
| 02389169 | Saxagliptin & Metformin | Komboglyze 2.5mg & 500mg Tab |
| 02389177 | Saxagliptin & Metformin | Komboglyze 2.5mg & 850mg Tab |
| 02389185 | Saxagliptin & Metformin | Komboglyze 2.5mg & 1000mg Tab |
| 02456575 | Empagliflozin & Metformin | Synjardy 5mg & 500mg Tab |
| 02456583 | Empagliflozin & Metformin | Synjardy 5mg & 850mg Tab |
| 02456591 | Empagliflozin & Metformin | Synjardy 5mg & 1000mg Tab |
| 02456605 | Empagliflozin & Metformin | Synjardy 12.5mg & 500mg Tab |
| 02456613 | Empagliflozin & Metformin | Synjardy 12.5mg & 850mg Tab |
| 02456621 | Empagliflozin & Metformin | Synjardy 12.5mg & 1000mg Tab |
| 02449935 | Dapagliflozin & Metformin | Xigduo 5mg & 850mg Tab |
| 02449943 | Dapagliflozin & Metformin | Xigduo 5mg & 1000mg Tab |
| 02257734 | Metformin HCL | Act Metformin (Off-Formulary Interchangeable) 850mg Tab |
| 02229785 | Metformin HCL | Apo-Metformin (Off-Formulary Interchangeable) 850mg Tab |
| 02305062 | Metformin HCL | Apo-Metformin ER (Off-Formulary Interchangeable) 500mg ER Tab |
| 02460653 | Metformin HCL | Apo-Metformin ER (Off-Formulary Interchangeable) 1000mg ER Tab |
| 02438283 | Metformin HCL | Auro-Metformin (Off-Formulary Interchangeable) 850mg Tab |
| 02421836 | Metformin HCL | Ecl-Metformin (Off-Formulary Interchangeable) 850mg Tab |
| 02380218 | Metformin HCL | Jamp-Metformin (Off-Formulary Interchangeable) 850mg Tab |
| 02380730 | Metformin HCL | Jamp-Metformin Blackberry (Off-Formulary Interchangeable) 850mg Tab |
| 02353385 | Metformin HCL | Metformin (Off-Formulary Interchangeable) 850mg Tab |
| 02385368 | Metformin HCL | Metformin FC (Off-Formulary Interchangeable) 850mg Tab |
| 02388774 | Metformin HCL | Mint-Metformin (Off-Formulary Interchangeable) 850mg Tab |
| 02242589 | Metformin HCL | PMS-Metformin (Off-Formulary Interchangeable) 850mg Tab |
| 02520311 | Metformin HCL | PMSC-Metformin (Off-Formulary Interchangeable) 850mg Tab |
| 02269058 | Metformin HCL | Ran-Metformin (Off-Formulary Interchangeable) 850mg Tab |
| 02242931 | Metformin HCL | Ratio-Metformin (Off-Formulary Interchangeable) 850mg Tab |
| 02246821 | Metformin HCL | Sandoz Metformin FC (Off-Formulary Interchangeable) 850mg Tab |
| 02162849 | Metformin HCL | Glucophage (Off-Formulary Interchangeable) 850mg Tab |
| 02268493 | Metformin HCL | Glumetza (Off-Formulary Interchangeable) 500mg ER Tab |
| 02300451 | Metformin HCL | Glumetza (Off-Formulary Interchangeable) 1000mg ER Tab |
| 02087324 | Bromocriptine | Bromocriptine 2.5mg Tab |
| 02230454 | Bromocriptine | Bromocriptine 5mg Cap |
| 00371033 | Bromocriptine | Parlodel (Not a Benefit) 2.5mg Tab |
| 00568643 | Bromocriptine | Parlodel (Not a Benefit) 5mg Cap |
| 02370921 | Linagliptin | Trajenta 5mg Tab |
| 02507471 | Saxagliptin | Apo-Saxagliptin 2.5mg Tab |
| 02507498 | Saxagliptin | Apo-Saxagliptin 5mg Tab |
| 02468603 | Saxagliptin | Sandoz Saxagliptin 2.5mg Tab |
| 02468611 | Saxagliptin | Sandoz Saxagliptin 5mg Tab |
| 02375842 | Saxagliptin | Onglyza 2.5mg Tab |
| 02333554 | Saxagliptin | Onglyza 5mg Tab |
| 02388839 | Sitagliptin Phosphate Monohydrate | Januvia 25mg Tab |
| 02388847 | Sitagliptin Phosphate Monohydrate | Januvia 50mg Tab |
| 02303922 | Sitagliptin Phosphate Monohydrate | Januvia 100mg Tab |
| 02471469 | Semaglutide | Ozempic 1.34mg/mL Inj Sol-Pref Pen 3mL Pk |
| 02471477 | Semaglutide | Ozempic 1.34mg/mL Inj Sol-Pref Pen 1.5mL Pk |
| 02355663 | Repaglinide | Apo-Repaglinide (Off-Formulary Interchangeable) 0.5mg Tab |
| 02355671 | Repaglinide | Apo-Repaglinide (Off-Formulary Interchangeable) 1mg Tab |
| 02355698 | Repaglinide | Apo-Repaglinide (Off-Formulary Interchangeable) 2mg Tab |
| 02424258 | Repaglinide | Auro-Repaglinide (Off-Formulary Interchangeable) 0.5mg Tab |
| 02424266 | Repaglinide | Auro-Repaglinide (Off-Formulary Interchangeable) 1mg Tab |
| 02424274 | Repaglinide | Auro-Repaglinide (Off-Formulary Interchangeable) 2mg Tab |
| 02321475 | Repaglinide | Co Repaglinide (Off-Formulary Interchangeable) 0.5mg Tab |
| 02321483 | Repaglinide | Co Repaglinide (Off-Formulary Interchangeable) 1mg Tab |
| 02321491 | Repaglinide | Co Repaglinide (Off-Formulary Interchangeable) 2mg Tab |
| 02354926 | Repaglinide | Jamp Repaglinide (Off-Formulary Interchangeable) 0.5mg Tab |
| 02354934 | Repaglinide | Jamp Repaglinide (Off-Formulary Interchangeable) 1mg Tab |
| 02354942 | Repaglinide | Jamp Repaglinide (Off-Formulary Interchangeable) 2mg Tab |
| 02357453 | Repaglinide | Sandoz Repaglinide (Off-Formulary Interchangeable) 0.5mg Tab |
| 02357461 | Repaglinide | Sandoz Repaglinide (Off-Formulary Interchangeable) 1mg Tab |
| 02357488 | Repaglinide | Sandoz Repaglinide (Off-Formulary Interchangeable) 2mg Tab |
| 02239924 | Repaglinide | GlucoNorm (Off-Formulary Interchangeable) 0.5mg Tab |
| 02239925 | Repaglinide | GlucoNorm (Off-Formulary Interchangeable) 1mg Tab |
| 02239926 | Repaglinide | GlucoNorm (Off-Formulary Interchangeable) 2mg Tab |
| 02435462 | Dapagliflozin | Forxiga 5mg Tab |
| 02435470 | Dapagliflozin | Forxiga 10mg Tab |
| 02425483 | Canagliflozin | Invokana 100mg Tab |
| 02425491 | Canagliflozin | Invokana 300mg Tab |
| 02443937 | Empagliflozin | Jardiance 10mg Tab |
| 02443945 | Empagliflozin | Jardiance 25mg Tab |
| 02269589 | Glimepiride | Sandoz Glimepiride (Off-Formulary Interchangeable) 1mg Tab |
| 02269597 | Glimepiride | Sandoz Glimepiride (Off-Formulary Interchangeable) 2mg Tab |
| 02269619 | Glimepiride | Sandoz Glimepiride (Off-Formulary Interchangeable) 4mg Tab |
| 02245272 | Glimepiride | Amaryl (Off-Formulary Interchangeable) 1mg Tab |
| 02245273 | Glimepiride | Amaryl (Off-Formulary Interchangeable) 2mg Tab |
| 02245274 | Glimepiride | Amaryl (Off-Formulary Interchangeable) 4mg Tab |
| 02429764 | Gliclazide | Act Gliclazide MR 30mg SR Tab |
| 02245247 | Gliclazide | Apo-Gliclazide 80mg Tab |
| 02297795 | Gliclazide | Apo-Gliclazide MR 30mg SR Tab |
| 02407124 | Gliclazide | Apo-Gliclazide MR 60mg ER Tab |
| 02287072 | Gliclazide | Gliclazide 80mg Tab |
| 02423286 | Gliclazide | Mint-Gliclazide MR 30mg SR Tab |
| 02423294 | Gliclazide | Mint-Gliclazide MR 60mg ER Tab |
| 02438658 | Gliclazide | Mylan-Gliclazide MR 30mg SR Tab |
| 02461323 | Gliclazide | Sandoz Gliclazide MR 30mg SR Tab |
| 02461331 | Gliclazide | Sandoz Gliclazide MR 60mg ER Tab |
| 02463571 | Gliclazide | Taro-Gliclazide MR 30mg SR Tab |
| 02439328 | Gliclazide | Taro-Gliclazide MR 60mg ER Tab |
| 02238103 | Gliclazide | Teva-Gliclazide 80mg Tab |
| 00765996 | Gliclazide | Diamicron 80mg Tab |
| 02242987 | Gliclazide | Diamicron MR 30mg SR Tab |
| 02356422 | Gliclazide | Diamicron MR 60mg ER Tab |
| 01913654 | Glyburide | Apo-Glyburide 2.5mg Tab |
| 02350459 | Glyburide | Glyburide 2.5mg Tab |
| 02350467 | Glyburide | Glyburide 5mg Tab |
| 02248008 | Glyburide | Sandoz Glyburide 2.5mg Tab |
| 01913670 | Glyburide | Teva-Glyburide 2.5mg Tab |
| 01913662 | Glyburide | Apo-Glyburide 5mg Tab |
| 02236734 | Glyburide | PMS-Glyburide 5mg Tab |
| 01913689 | Glyburide | Teva-Glyburide 5mg Tab |
| 02224550 | Glyburide | Diabeta (Not a Benefit) 2.5mg Tab |
| 02224569 | Glyburide | Diabeta (Not a Benefit) 5mg Tab |
| 02403366 | Rosiglitazone | Rosiglitazone (Off-Formulary Interchangeable) 2mg Tab |
| 02403374 | Rosiglitazone | Rosiglitazone (Off-Formulary Interchangeable) 4mg Tab |
| 02403382 | Rosiglitazone | Rosiglitazone (Off-Formulary Interchangeable) 8mg Tab |
| 02241112 | Rosiglitazone | Avandia (Off-Formulary Interchangeable) 2mg Tab |
| 02241113 | Rosiglitazone | Avandia (Off-Formulary Interchangeable) 4mg Tab |
| 02241114 | Rosiglitazone | Avandia (Off-Formulary Interchangeable) 8mg Tab |
| 02303442 | Pioglitazone Hcl | Accel Pioglitazone (Off-Formulary Interchangeable) 15mg Tab |
| 02303450 | Pioglitazone Hcl | Accel Pioglitazone (Off-Formulary Interchangeable) 30mg Tab |
| 02303469 | Pioglitazone Hcl | Accel Pioglitazone (Off-Formulary Interchangeable) 45mg Tab |
| 02302861 | Pioglitazone Hcl | Act Pioglitazone (Off-Formulary Interchangeable) 15mg Tab |
| 02302888 | Pioglitazone Hcl | Act Pioglitazone (Off-Formulary Interchangeable) 30mg Tab |
| 02302896 | Pioglitazone Hcl | Act Pioglitazone (Off-Formulary Interchangeable) 45mg Tab |
| 02302942 | Pioglitazone Hcl | Apo-Pioglitazone (Off-Formulary Interchangeable) 15mg Tab |
| 02302950 | Pioglitazone Hcl | Apo-Pioglitazone (Off-Formulary Interchangeable) 30mg Tab |
| 02302977 | Pioglitazone Hcl | Apo-Pioglitazone (Off-Formulary Interchangeable) 45mg Tab |
| 02384906 | Pioglitazone Hcl | Auro-Pioglitazone (Off-Formulary Interchangeable) 15mg Tab |
| 02384914 | Pioglitazone Hcl | Auro-Pioglitazone (Off-Formulary Interchangeable) 30mg Tab |
| 02384922 | Pioglitazone Hcl | Auro-Pioglitazone (Off-Formulary Interchangeable) 45mg Tab |
| 02397307 | Pioglitazone Hcl | Jamp-Pioglitazone (Off-Formulary Interchangeable) 15mg Tab |
| 02365529 | Pioglitazone Hcl | Jamp-Pioglitazone (Off-Formulary Interchangeable) 30mg Tab |
| 02365537 | Pioglitazone Hcl | Jamp-Pioglitazone (Off-Formulary Interchangeable) 45mg Tab |
| 02326477 | Pioglitazone Hcl | Mint-Pioglitazone (Off-Formulary Interchangeable) 15mg Tab |
| 02326485 | Pioglitazone Hcl | Mint-Pioglitazone (Off-Formulary Interchangeable) 30mg Tab |
| 02326493 | Pioglitazone Hcl | Mint-Pioglitazone (Off-Formulary Interchangeable) 45mg Tab |
| 02303124 | Pioglitazone Hcl | PMS-Pioglitazone (Off-Formulary Interchangeable) 15mg Tab |
| 02303132 | Pioglitazone Hcl | PMS-Pioglitazone (Off-Formulary Interchangeable) 30mg Tab |
| 02303140 | Pioglitazone Hcl | PMS-Pioglitazone (Off-Formulary Interchangeable) 45mg Tab |
| 02391600 | Pioglitazone Hcl | Pioglitazone Hydrochloride Tablets (Off-Formulary Interchangeable) 15mg Tab |
| 02339587 | Pioglitazone Hcl | Pioglitazone Hydrochloride Tablets (Off-Formulary Interchangeable) 30mg Tab |
| 02339595 | Pioglitazone Hcl | Pioglitazone Hydrochloride Tablets (Off-Formulary Interchangeable) 45mg Tab |
| 02297906 | Pioglitazone Hcl | Sandoz Pioglitazone (Off-Formulary Interchangeable) 15mg Tab |
| 02297914 | Pioglitazone Hcl | Sandoz Pioglitazone (Off-Formulary Interchangeable) 30mg Tab |
| 02297922 | Pioglitazone Hcl | Sandoz Pioglitazone (Off-Formulary Interchangeable) 45mg Tab |
| 02274914 | Pioglitazone Hcl | Teva-Pioglitazone (Off-Formulary Interchangeable) 15mg Tab |
| 02274922 | Pioglitazone Hcl | Teva-Pioglitazone (Off-Formulary Interchangeable) 30mg Tab |
| 02274930 | Pioglitazone Hcl | Teva-Pioglitazone (Off-Formulary Interchangeable) 45mg Tab |
| 02242572 | Pioglitazone Hcl | Actos (Off-Formulary Interchangeable) 15mg Tab |
| 02242573 | Pioglitazone Hcl | Actos (Off-Formulary Interchangeable) 30mg Tab |
| 02242574 | Pioglitazone Hcl | Actos (Off-Formulary Interchangeable) 45mg Tab |
| Drug Identification Numbers (DIN) used to classify a patient as having diabetes. | | |

# Table S4: Characteristics of repairs by joint (entire cohort)

|  | **Hip**  **(n=204,066)** | **Knee**  **(n=341,678)** |
| --- | --- | --- |
| Patient demographics |  |  |
| Age (years) | 67.7 (SD 11.3) | 68.2 (SD 9.2) |
|  |  |  |
| Sex |  |  |
| Female | 111,272 (55%) | 208,751 (61%) |
| Male | 92,794 (45%) | 132,927 (39%) |
|  |  |  |
| Procedure characteristics |  |  |
| Setting |  |  |
| Inpatient | 186,531 (91%) | 319,356 (93%) |
| Outpatient | 17,535 (8.6%) | 22,322 (6.5%) |
|  |  |  |
| Replacement order – original |  |  |
| Primary | 190671 (93%) | 324,408 (95%) |
| Revision | 13395 (6.6%) | 17,270 (5.1%) |
|  |  |  |
| Replacement order – modified^a^ |  |  |
| Primary | 141,907 (70%) | 228,518 (67%) |
| Revision | 62,159 (30%) | 113,160 (33%) |
|  |  |  |
| Replacement type |  |  |
| Total | 198,883 (97%) | 334,885 (98%) |
| Partial | 5,183 (2.5%) | 6,793 (2.0%) |
|  |  |  |
| Laterality |  |  |
| Unilateral | 202,720 (99%) | 334,755 (98%) |
| Bilateral | 1,346 (0.7%) | 6,923 (2.0%) |
|  |  |  |
| Clinical characteristics |  |  |
| Charlson Comorbidity score |  |  |
| 0 (none) | 144,347 (71%) | 222,731 (65%) |
| 1 | 40,000 (20%) | 81,353 (24%) |
| 2 | 12,781 (6.3%) | 25,442 (75%) |
| 3+ | 6,938 (3.4%) | 12,152 (3.6%) |
|  |  |  |
| Most responsible diagnosis |  |  |
| Osteoarthritis | 183,425 (90%) | 322,821 (94%) |
| Trauma | 1,053 (0.5%) | 77 (<0.1%) |
| Mechanical complication of prosthesis | 10,922 (5.4%) | 12,318 (3.6%) |
| Infection/inflammation d/t prosthesis | 2,375 (1.2%) | 3,281 (1.0%) |
| Osteonecrosis | 3,692 (1.8%) | 288 (0.1%) |
| Cancer | 428 (0.2%) | 62 (<0.1%) |
| Non-traumatic fracture (e.g. pathological, nonunion, malunion) | 657 (0.3%) | 97 (<0.1%) |
| Arthritis, other or NOS | 793 (0.4%) | 1,169 (0.3%) |
| Rheumatoid arthritis | 677 (0.3%) | 1,553 (0.5%) |
| Missing | 44 | 12 |
|  |  |  |
| Patient socio-demographics |  |  |
| Rurality |  |  |
| Urban | 168,786 (83%) | 284,922 (84%) |
| Rural | 33,910 (17%) | 54,516 (16%) |
| Missing | 1370 (<1%) | 2240 (<1%) |
|  |  |  |
| Deprivation |  |  |
| 1 (least marginalized) | 50,334 (25%) | 72,670 (22%) |
| 2 | 42,931 (21%) | 69,737 (21%) |
| 3 | 39,281 (20%) | 67,897 (20%) |
| 4 | 36,488 (18%) | 65,984 (20%) |
| 5 (most marginalized) | 31,405 (16%) | 59,316 (18%) |
| Missing | 3627 (2%) | 6074 (2%) |
|  |  |  |
| Instability |  |  |
| 1 (least marginalized) | 29,549 (15%) | 55,144 (16%) |
| 2 | 40,565 (20%) | 67,307 (20%) |
| 3 | 43,068 (22%) | 72,194 (22%) |
| 4 | 40,794 (20%) | 67,749 (20%) |
| 5 (most marginalized) | 46,463 (23%) | 73,210 (22%) |
| Missing | 3627 (2%) | 6074 (2%) |
|  |  |  |
| Dependency |  |  |
| 1 (least marginalized) | 27,640 (14%) | 50,034 (15%) |
| 2 | 31,735 (16%) | 53,935 (16%) |
| 3 | 35,427 (18%) | 58,614 (17%) |
| 4 | 39,058 (19%) | 64,527 (19%) |
| 5 (most marginalized) | 66,579 (33%) | 108,494 (32%) |
| Missing | 3627 (2%) | 6074 (2%) |
|  |  |  |
| Ethnic diversity |  |  |
| 1 (least diverse) | 54,848 (27%) | 89,287 (27%) |
| 2 | 46,852 (23%) | 74,099 (22%) |
| 3 | 41,113 (21%) | 62,882 (19%) |
| 4 | 34,550 (17%) | 54,513 (16%) |
| 5 (most diverse) | 23,076 (12%) | 54,823 (16%) |
| Missing | 3627 (2%) | 6074 (2%) |
| ^a^ reclassified as a revision if there was evidence of hardware removal or a prior ipsilateral arthroplasty | | |

# Table S5: Sensitivity analysis by statistical model for 90-day outcomes

|  | **Hip arthroplasty** | | | | **Knee arthroplasty** | | | |  |
| --- | --- | --- | --- | --- | --- | --- | --- | --- | --- |
|  | Inpatient (n=24,596) | Outpatient  (n=6,969) |  | Inpatient  (n=37,473) | | Outpatient  (n=9,334) |  |  |  |
| **Readmissions (90-day)^a,b^** |  | RR (95% CI) | p-value |  | | RR (95% CI) | p-value |  |  |
| Crude (Table 2) | 1.0 (ref) | 0.48 (0.42-0.55) | <.0001 | 1.0 (ref) | | 0.71 (0.63-0.79) | <.0001 |  |  |
| Adjusted^c^ | 1.0 (ref) | 0.66 (0.58-0.76) | <.0001 | 1.0 (ref) | | 0.87 (0.77-0.97) | 0.01 |  |  |
| Matched crude (Table 2) | 1.0 (ref) | 0.65 (0.56-0.76) | <.0001 | 1.0 (ref) | | 0.92 (0.88-0.96) | 0.0005 |  |  |
| Matched and adjusted | 1.0 (ref) | 0.66 (0.55-0.78) | <.0001 | 1.0 (ref) | | 0.92 (0.88-0.97) | 0.0007 |  |  |
|  |  |  |  |  | |  |  |  |  |
| **Unplanned ED visits (90-day)^a,b^** |  | **RR (95% CI)** | **p-value** |  | | **RR (95% CI)** | **p-value** |  |  |
| Crude (Table 2) | 1.0 (ref) | 0.72 (0.68-0.76) | <.0001 | 1.0 (ref) | | 0.85 (0.82-0.89) | <.0001 |  |  |
| Adjusted | 1.0 (ref) | 0.80 (0.76-0.85) | <.0001 | 1.0 (ref) | | 0.92 (0.88-0.96) | <.0001 |  |  |
| Matched crude (Table 2) | 1.0 (ref) | 0.78 (0.73-0.83) | <.0001 | 1.0 (ref) | | 0.86 (0.76-0.97) | 0.01 |  |  |
| Matched and adjusted | 1.0 (ref) | 0.78 (0.72-0.84) | <.0001 | 1.0 (ref) | | 0.87 (0.77-0.98) | 0.02 |  |  |
|  |  |  |  |  | |  |  |  |  |
| **1-year composite event^d^** |  | **RR (95% CI)** | **p-value** |  | | **RR (95% CI)** | **p-value** |  |  |
| Crude (Table 2) | 1.0 (ref) | 0.45 (0.32-0.63) | <.0001 | 1.0 (ref) | | 0.74 (0.55-0.99) | 0.04 |  |  |
| Adjusted | 1.0 (ref) | 0.69 (0.49-0.98) | 0.04 | 1.0 (ref) | | 0.95 (0.69-1.30) | 0.74 |  |  |
| Matched crude (Table 2) | 1.0 (ref) | 0.65 (0.45-0.93) | 0.02 | 1.0 (ref) | | 0.90 (0.64-1.26) | 0.54 |  |  |
| Matched and adjusted | 1.0 (ref) | 0.64 (0.42-0.97) | 0.03 | 1.0 (ref) | | 0.92 (0.66-1.29) | 0.64 |  |  |
|  |  |  |  |  | |  |  |  |  |
| Risk ratio (RR) and confidence interval (CI) comparing outpatient versus inpatient on readmissions, unplanned emergency department visits, and 1-year event following hip or knee arthroplasty comparing different statistical models: 1) crude; 2) adjusted; 3) propensity-score-matched, crude; and 4) propensity-score matched, adjusted.  ^a^ Readmission or emergency department (ED) visit within 90 days or 1 year of discharge (inpatient) or registration date (outpatient) following primary arthroplasty  ^b^ primary arthroplasties occurred between March 2020 and September 2022  ^c^ adjusted for age, sex, comorbidity score, partial/total arthroplasty, rurality, material deprivation quintile, dependency quintile, residential instability quintile, and ethnic diversity quintile  ^d^ primary arthroplasties occurred between March 2020 and December 2021 (for 1-year follow-up) | | | | | | | | | |
