# Supplementary material for: The successful and safe conversion of joint arthroplasty to same-day surgery: A necessity after the COVID-19 pandemic
Source: PLoS One. 2023 Nov 27;18(11):e0290135. doi: 10.1371/journal.pone.0290135 (PMC10681212; doi:10.1371/journal.pone.0290135)
Supplement: Attachment — Submitted filename: Response to reviewers - Hip Knee.docx [file pone.0290135.s002.docx]

October 18, 2023

**Re:** PONE-D-23-22900: The Impact of the COVID-19 Pandemic on Outpatient Hip and Knee Arthroplasty in Ontario, Canada

Dear Dr Huang,

On behalf of the co-authors, we thank you and the reviewers for the review and suggestions to improve the manuscript. We provide point-by-point responses below. No changes were made to our conflicts of interest or the financial discloser, and for convenience, the original cover letter is included below. Regarding access to data, we are not permitted to share data publicly for legal and privacy reasons, so I believe our data availability statement is appropriate.

Thank you for your time and consideration

Sincerely,

Steven Habbous, PhD

Epidemiologist

Ontario Health (Strategic Analytics)

Email: [steven.habbous@ontariohealth.on.ca](mailto:steven.habbous@ontariohealth.on.ca)

2-Oct-2023

**Re:** PONE-D-23-22900: The Impact of the COVID-19 Pandemic on Outpatient Hip and Knee Arthroplasty in Ontario, Canada

Dear Dr Habbous,

Thank you for submitting your manuscript to PLOS ONE. After careful consideration, we feel that it has merit but does not fully meet PLOS ONE’s publication criteria as it currently stands. Therefore, we invite you to submit a revised version of the manuscript that addresses the points raised during the review process.

Please submit your revised manuscript by Nov 16 2023 11:59PM. If you will need more time than this to complete your revisions, please reply to this message or contact the journal office at [plosone@plos.org](mailto:plosone@plos.org). When you're ready to submit your revision, log on to https://www.editorialmanager.com/pone/ and select the 'Submissions Needing Revision' folder to locate your manuscript file.

Please include the following items when submitting your revised manuscript:

- A rebuttal letter that responds to each point raised by the academic editor and reviewer(s). You should upload this letter as a separate file labeled 'Response to Reviewers'.
- A marked-up copy of your manuscript that highlights changes made to the original version. You should upload this as a separate file labeled 'Revised Manuscript with Track Changes'.
- An unmarked version of your revised paper without tracked changes. You should upload this as a separate file labeled 'Manuscript'.

If you would like to make changes to your financial disclosure, please include your updated statement in your cover letter. Guidelines for resubmitting your figure files are available below the reviewer comments at the end of this letter.

If applicable, we recommend that you deposit your laboratory protocols in protocols.io to enhance the reproducibility of your results. Protocols.io assigns your protocol its own identifier (DOI) so that it can be cited independently in the future. For instructions see: <https://journals.plos.org/plosone/s/submission-guidelines#loc-laboratory-protocols>. Additionally, PLOS ONE offers an option for publishing peer-reviewed Lab Protocol articles, which describe protocols hosted on protocols.io. Read more information on sharing protocols at <https://plos.org/protocols?utm_medium=editorial-email&utm_source=authorletters&utm_campaign=protocols>.

We look forward to receiving your revised manuscript.

Kind regards,

Kuo-Cherh Huang
Academic Editor
PLOS ONE

July 20, 2023

Re: Outpatient hip & knee arthroplasty

Dear editor,

On behalf of our co-authors, we would like to submit our original article entitled “**The Impact of the COVID-19 Pandemic on Outpatient Hip and Knee Arthroplasty in Ontario, Canada**” for consideration in your journal.

**Data availability:** Ontario Health is prohibited from making the data used in this research publicly accessible if it includes potentially identifiable personal health information and/or personal information as defined in Ontario law, specifically the Personal Health Information Protection Act (PHIPA) and the Freedom of Information and Protection of Privacy Act (FIPPA). Due to these legal and ethical restrictions, data will not be made publicly available. However, upon request, data de-identified to a level suitable for public release may be provided.

**Billing:** Steven Habbous (lead and corresponding author) is affiliated with Western University, Ontario, Canada. Given the relationship between Western University and PLoS journals, the university can be billed directly if accepted for publication.

**Brief overview:** The COVID-19 pandemic has resulted in a marked reduction in elective hip and knee arthroplasty. However, this has also resulted in a shift from a predominantly inpatient procedure (1% pre-pandemic) to 39% (hip) and 36% (knee) by 2022. We demonstrate significant regional variability in the uptake of outpatient arthroplasty. After propensity-score matching, the rate of 90-day readmissions, 90-day emergency department visits, and 1-year composite of mortality, infection, or revision was not different or better following outpatient compared with inpatient arthroplasty. Outpatient hip and knee replacement has the potential to remediate the surgical backlog caused by the pandemic and is likely here to stay.

The contents of this manuscript are not currently under consideration for publication elsewhere. All authors of this research paper participated in the preparation of this manuscript and any conflicts of interest are detailed in the manuscript. On behalf of the authors, thank you in advance for your time and consideration.

Sincerely,

Steven Habbous, PhD

Epidemiologist

Ontario Health (Strategic Analytics)

Email: [steven.habbous@ontariohealth.on.ca](mailto:steven.habbous@ontariohealth.on.ca)

Journal requirements:

When submitting your revision, we need you to address these additional requirements.

1. Please ensure that your manuscript meets PLOS ONE's style requirements, including those for file naming. The PLOS ONE style templates can be found at

https://journals.plos.org/plosone/s/file?id=wjVg/PLOSOne_formatting_sample_main_body.pdf and

https://journals.plos.org/plosone/s/file?id=ba62/PLOSOne_formatting_sample_title_authors_affiliations.pdf

2. We note that you have indicated that data from this study are available upon request. PLOS only allows data to be available upon request if there are legal or ethical restrictions on sharing data publicly. For more information on unacceptable data access restrictions, please see http://journals.plos.org/plosone/s/data-availability#loc-unacceptable-data-access-restrictions.

In your revised cover letter, please address the following prompts:

a) If there are ethical or legal restrictions on sharing a de-identified data set, please explain them in detail (e.g., data contain potentially sensitive information, data are owned by a third-party organization, etc.) and who has imposed them (e.g., an ethics committee). Please also provide contact information for a data access committee, ethics committee, or other institutional body to which data requests may be sent.

b) If there are no restrictions, please upload the minimal anonymized data set necessary to replicate your study findings as either Supporting Information files or to a stable, public repository and provide us with the relevant URLs, DOIs, or accession numbers. For a list of acceptable repositories, please see http://journals.plos.org/plosone/s/data-availability#loc-recommended-repositories.

We will update your Data Availability statement on your behalf to reflect the information you provide.

3. Please review your reference list to ensure that it is complete and correct. If you have cited papers that have been retracted, please include the rationale for doing so in the manuscript text, or remove these references and replace them with relevant current references. Any changes to the reference list should be mentioned in the rebuttal letter that accompanies your revised manuscript. If you need to cite a retracted article, indicate the article’s retracted status in the References list and also include a citation and full reference for the retraction notice.

**EDITOR COMMENTS:**

Additional Editor Comments:

Dear Dr. Habbous,

We appreciate your submission to PLoS ONE. Both referees had provided a variety of comments on your manuscript. Here, I would like to bring up a couple of points. Firstly, please consider the nice suggestion from Reviewer 1 in regard to the manuscript title. Secondly, both reviewers had made inquiries and provided suggestions concerning the Methods part. Please respond to them thoughtfully, and explain where you feel you cannot completely agree with reviewers’ suggestions. Finally, I observed that Reviewer 2 had suggested an explicit reference for you to cite. You may wish to consider if the suggested citation is appropriate to be included if the paper fits within your current work. I would like to emphasize that the odds of the acceptance of your submission will not depend on your decision in respect of the suggested citation, however. Thank you.

Academic Editor
Kuo-Cherh Huang

**Comments to the Author**

**REVIEWER COMMENTS:**

**Reviewer: 1**
I enjoyed the paper and I think it makes a strong argument for the progression to same day surgery. But I would like to make some suggestions.

**Comment #1:** TITLE - The Impact of the COVID-19 Pandemic on Outpatient Hip and Knee Arthroplasty in Ontario, Canada

I wonder if the title should be more of a "headline" rather than what it is now? You've shown that the pandemic was a huge catalyst for change. And that much of the province HAD to pivot to same day surgery; and that the change was successful with no increase in complication or readmissions.
"The Covid-19 Pandemic: a catalyst to same day arthroplasty across a Province, with no increase in complications or readmissions" This title is just a suggestion, but maybe it grabs me more and makes me feel that I'd want to read it.

or another example..
"The successful and safe in conversion of joint arthroplasty to same day surgery: a Necessity after the covid pandemic".

or maybe even highlight the fact that this conversion occurred in a universal healthcare system as well?

**Response:** These are great suggestions. The transition to outpatient arthroplasty was certainly encouraged by the pandemic. We changed the title to “The successful and safe conversion of joint arthroplasty to same-day surgery: a necessity after the COVID-19 pandemic”

**Comment #2:** Methodology
I am aware of the healthcare across Canada and in particular the Universal Healthcare system. In your methodology, I'm not sure you express how robust the universality is in capturing data. I have read other articles that show this more clearly - and this is the strength of your paper. The fact that you have a single insurer and virtually all hospital and clinic visits are captured by ICES, the DAD and CCI.
This means that your data is capturing virtually all of the half million cases and then virtually all the follow up visits. This is such a unique system and dataset that is not really available elsewhere. I would sing this from the rooftop if it was available to me.

**Response:** Thank you, we are certainly privileged to have access to such comprehensive population-based data. We added the following statements to the methods to describe our data sources more fulsomely:

“All hospitals in Ontario are mandated to report all admission records (discharge abstracts) to the CIHI-DAD. Coding is performed by trained nosologists and routine data quality checks are performed (reference: <https://www.cihi.ca/sites/default/files/document/dad-data-quality-current-year-information-2021-2022-report-en.pdf>). All surgical outpatient hospital visits are reported to either the CIHI-DAD or CIHI-NACRS as a Level 3 submission, which mandates ICD-10-CA and CCI coding (<https://www.cihi.ca/sites/default/files/document/nacrs-data-quality-current-year-information-2021-2022-report-en.pdf>).”

**Comment #3:** Is this the largest series looking at the conversion of arthroplasty to same day surgery? If it is, then please highlight this too.

**Response:** Following a deeper dive of the existing literature, ours is the largest study to date comparing inpatient with outpatient hip/knee arthroplasty at the population-level from a universal health system. The only other population-based study arose from the United States, which operates under a different health care system. We have therefore added the following to the introduction:

“A systematic review of the literature comparing inpatient with outpatient hip and knee arthroplasty captured studies predominantly from the United States, and studies from other countries were sparse (two from Canada and three from Europe) with small sample sizes (<600).”

And the following statement as well:

“This is the largest population-based analyses to date from a universal health care system on this topic.”

**Comment #4:** Can you break down where these conversions happened most? i.e. was there a trend for it to occur in just teaching/ academic hospitals or was it even in community hospitals also?

**Response:** Interestingly, there was no trend one way or the other for what type of hospitals tended to perform more outpatient procedures. Some high-volume hospitals (e.g. typically academic) performed relative few outpatient procedures, while other performed more. Similarly for and community hospitals and small-volume hospitals, there was no obvious trend. Plotting this confirms this:

**Comment #5:** Overall, I found this paper very exciting and the message is that even in a universal healthcare system, hospitals had to shift their practices in order to keep wait times low. This will then show other nations/ healthcare providers there are potentials for the same.

This is where the importance of this paper is.

**Response:** Thank you for your review!

**Reviewer: 2**

This research analyzes how the provision of outpatient hip and knee arthroplasty in Ontario has changed over time, with a focus on the COVID-19 pandemic period.
The abstract is structured.

**Comment #1:** The introduction is short and does not achieve a sufficiently good transposition in the topic of the problem. It would be recommended to refer to similar studies from other countries to emphasize the importance of the topic. As a consequence, the hypotheses of the study should be formulated.

**Response:** Thank you for raising this point, we agree. After a more thorough review of the literature, we have added the following statement to the introduction to highlight the dearth of evidence on this topic from a population-level:

“…the Centers for Medicare and Medicaid Services in the United States removed from the inpatient-only list total knee replacement in 2018 and total hip replacement in 2020, resulting in a drastic shift towards outpatient arthroplasty.(PMID 36535441, 36898484) Emergency department visits and readmissions were favourable following outpatient hip but worse following outpatient knee replacement. A systematic review of the literature comparing inpatient with outpatient hip and knee arthroplasty captured studies predominantly from the United States, and studies from other countries were sparse (two from Canada and three from Europe) and not population-based (study size <600)…This is the largest population-based analyses to date from a universal health care system on this topic.”

**Comment #2:** The formulated objective does not detail the analysis period.

**Response:** The final sentence of the introduction that sites the objective now indicates the study period. As this was an exploratory study, a time period of interest was not pre-specified. Rather, we included historical data as far back as our data permits (2010) while enabling adjustment for covariates like comorbidity that have a 3-year look-back period. We add this reason to the methods.

**Comment #3:** The research methodology should briefly describe at the beginning the steps followed and then detail them.

**Response:** We added the following preamble at the beginning of the methods:

“In this retrospective population-based cohort study, inpatient and outpatient hip and knee arthroplasty surgeries were captured between 2010 and 2022. We examined 1) the effect of the pandemic on surgical volumes; 2) trends of inpatient versus outpatient arthroplasty; and 3) outcomes comparing inpatient with outpatient arthroplasty.”

**Comment #4:** The significance of the volume of data analyzed should be studied.

**Response:** Following a deeper dive of the existing literature, ours is the largest study to date comparing inpatient with outpatient hip/knee arthroplasty at the population-level from a universal health system. The only other country with similar studies are from the United States, which operates under a different health care system. We have therefore added the following to the introduction:

“A systematic review of the literature comparing inpatient with outpatient hip and knee arthroplasty captured studies predominantly from the United States, and studies from other countries were sparse (two from Canada and three from Europe) with small sample sizes (<600)… This is the largest population-based analyses to date from a universal health care system on this topic.”

**Comment #5:** The discussions are conducted only in relation to the results of this study and do not refer to the results reported in other countries. A comparative recommendation is the recent study:
Moldovan F, Moldovan L, Bataga T. A Comprehensive Research on the Prevalence and Evolution Trend of Orthopedic Surgeries in Romania. Healthcare. 2023; 11(13):1866. DOI: 10.3390/healthcare11131866
But also other similar studies.

**Response:** Thank you for sharing this reference. That specific study from Romania forecasts an increasing number of hip/knee arthroplasties over the next decade, so perhaps the Romanian healthcare system can consider outpatient joint replacement for capacity planning (appears to be all inpatient). In this revision, we update the discussion with more recent evidence from the literature with no restriction on country of origin. A systematic review culling the literature until August 2021 identified studies predominantly from the United States (reference #7). The only studies from other countries were small: (n=86 and n=274 from Canada; n=574 from France; n=607 from Netherlands; n=455 from Denmark). A fresh look at the literature did not identify more recent studies except from the United States, which we now cite in the introduction. We added the following to the discussion, citing forecasting studies from the United States, Romania (your suggested article), Taiwan, and Australia:

“With aging populations, there is expectation that the number of hip and knee arthroplasty procedures will increase, and jurisdictions that still perform inpatient-only procedures may be forced to consider the outpatient setting.(PMID 30180053, 37444700, 26318767, 30797228)”

**Comment #6:** The conclusions should indicate the percentage of the monitored variables.

**Response:** We have added a “missing” row for Table 1 for variables with missing data. Fortunately the proportion of records with missing data were small (<2%) and non-differential by inpatient versus outpatient setting.
